# Antibody response elicited by a third boost dose of inactivated SARS-CoV-2 vaccine can neutralize SARS-CoV-2 variants of concern

**DOI:** 10.1080/22221751.2021.1996210

**Published:** 2021-11-16

**Authors:** Lei Yue, Jian Zhou, Yanan Zhou, Xiaolei Yang, Tianhong Xie, Mengli Yang, Hongling Zhao, Yuan Zhao, Ting Yang, Hua Li, Hong Xiang, Jie Wang, Shuaiyao Lu, Hongqi Liu, Hong Zhao, Xingchen Wei, Yuhao Zhang, Zhongping Xie

**Affiliations:** The Institute of Medical Biology, Chinese Academy of Medical Sciences and Peking Union Medical College, Kunming, People’s Republic of China

## Dear editor

Since the initial outbreak in late 2019, coronavirus disease 2019 (COVID-19), which is caused by severe acute respiratory syndrome coronavirus 2 (SARS-CoV-2) infection, has evolved into a global pandemic [1,2]. Inactivated vaccines [3], mRNA vaccines [4], and adenovirus vector vaccines [5] have been developed based on different platforms. Several vaccines have obtained emergency use authorization from the World Health Organization. Recently, the U.S. Food and Drug Administration approved the first COVID-19 vaccine (Pfizer-BioNTech COVID-19 Vaccine) [6]. Mass vaccination has played an important role in the effective control of COVID-19 epidemic worldwide [7].

Inactivated SARS-CoV-2 vaccines have been mainly developed by companies in developing countries, and clinical trials showed good safety profiles and protect against COVID-19 [3,8,9]. Inactivated vaccines have been approved by dozens of countries and jurisdictions [10]. Current research shows that 6 months after two doses of inactivated vaccine, the neutralizing antibody wanes significantly, although the immune memory is not disappeared [11]. Neutralizing antibody levels are highly predictive of immune protection from symptomatic SARS-CoV-2 infection [12]. Therefore, the necessity of the third booster dose is constant concerned. In addition, facing the constantly emerging variants of concern, it is still uncertain whether a booster dose of inactivated vaccine can evoke immune memory quickly to provide important protection.

**Figure 1.**
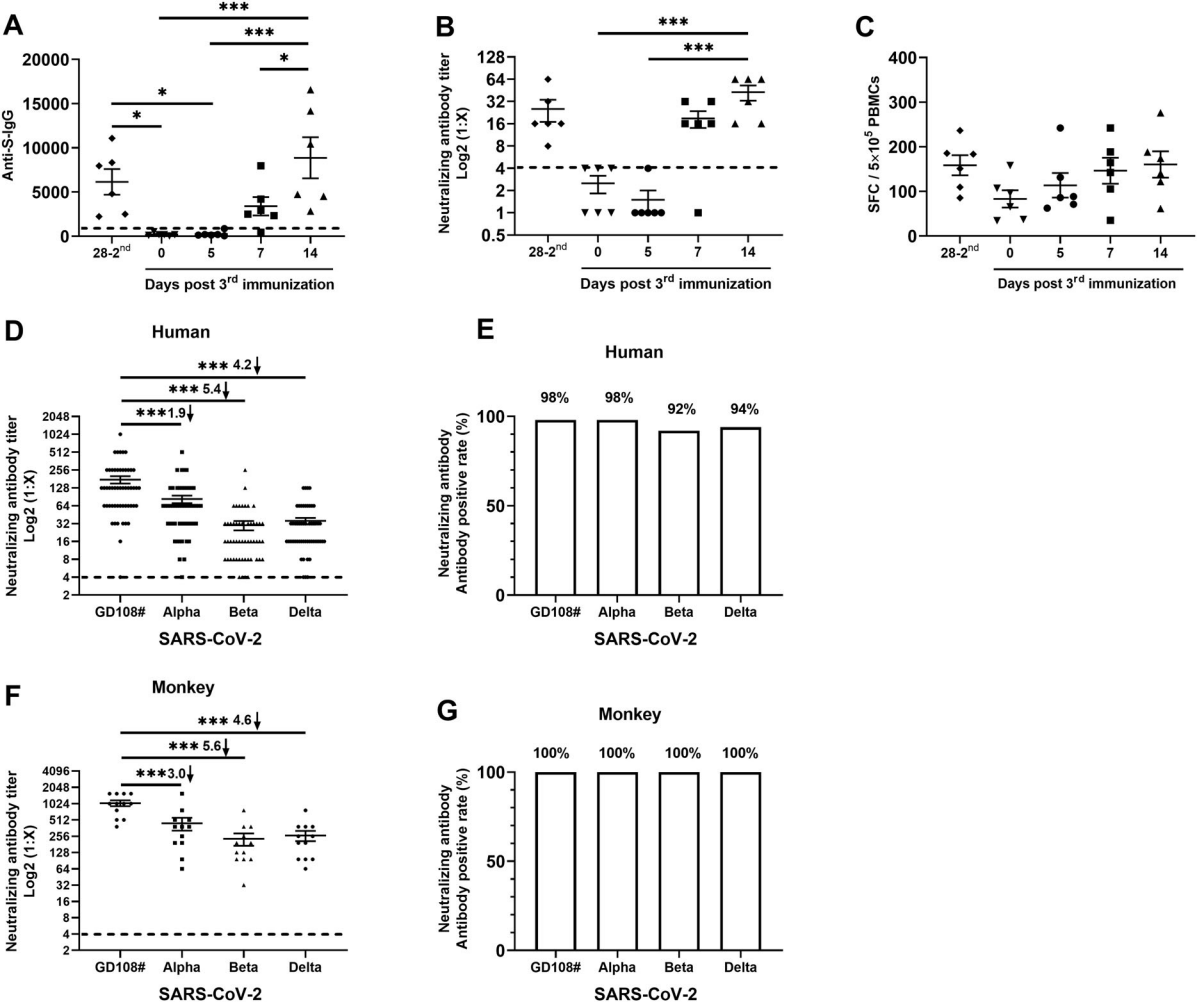
Antibody response elicited by a third boost dose of inactivated SARS-CoV-2 vaccine can neutralize SARS-CoV-2 variants of concern. (A–C) Enzyme-linked immunosorbent assay (ELISA) antibody against S protein, neutralizing antibody against original strain (GD108#), and the IFN-γ-specific T-cell responses against the S, N, M, and O antigens induced by a third booster dose of inactivated SARS-CoV-2 vaccine (*n* = 6). (D and E) Human neutralizing antibodies and positive rate against original strain (GD108#) and variants (alpha, beta, delta) induced by the inactivated SARS-CoV-2 vaccine 14 days after a third booster dose (*n* = 53). (F, G) Monkey neutralizing antibodies and positive rate against original strain (GD108#) and variants induced by the inactivated SARS-CoV-2 vaccine 14 days after a third booster dose (*n* = 12). The neutralizing antibody-positive judgment threshold is marked with a dotted line. (*p < 0.05; **p < 0.01; ***p < 0.001).

In this study, 53 volunteers, who joined in the development and production of inactivated vaccines (with informed consent), received two doses (at 0 and 28 days) of inactivated COVID-19 vaccines in 2020. Due to the need to further explore COVID-19 vaccines, they received a third dose 8 months after the second dose recently. At 0, 5, 7, and 14 days after the third dose, blood was collected from 6 volunteers for detection of anti-S IgG antibody (Figure 1A), neutralizing antibody titre (Figure 1B) and specific IFN-γ-secreting T-cell response (Figure 1C). We found that both the anti-S antibody and neutralizing antibody against the original strain (GD108#) gradually increased after 5 days, and the positive conversion rate of antibodies reached 100% at 14 days. Interestingly, the memory of IFN-γ-T cells against S, N, M, O antigens of SARS-CoV-2 can be quickly awakened after the third dose. These results indicate that although the neutralizing antibodies gradually decrease after two doses of inactivated vaccines, the antibody response could be awakened quickly and the T-cell immune memory is still active.

To address the question that whether a third booster dose could provide protection to variants of concern. Here, we assessed cross-protection capacity against alpha, beta and delta variants on 53 human sera and 12 monkey sera of 14 days after the third booster dose. It is encouraging that the neutralizing antibody can neutralize recently emerged SARS-CoV-2 variants, and the antibody-positive conversion rate exceeds 90%, even if the human neutralizing antibodies titre decreased approximately 1.9, 5.4, 4.2 times against alpha, beta and delta variants, respectively, and the monkey neutralizing antibodies decreased approximately 3.0, 5.6, 4.6 times (Figure 1D–G).

Our studies provided evidence for the efficacy of a third booster dose of inactivated SARS-CoV-2 vaccine against variants of concern. However, it is necessary to evaluate its effectiveness in the real world in the future. A recent real-world study conducted in Guangzhou (China) showed that the protection rate of two doses inactivated vaccine against delta variant infection exceeded 50% [13]. In addition, vaccination with CoronaVac was associated with a reduction in symptomatic Covid-19, hospital admissions, and deaths in adults aged ≥70 years in a setting with extensive transmission of the gamma variant in Brazil [10]. Given that the neutralizing antibody of 1 month after a third booster dose is significantly higher than that of a two-dose procedure [14], we believe that a three-dose procedure may be more effective against variants. Continuously observing the persistence of the protection provided by vaccines in real cases and the effectiveness of a third booster dose, conducting long-term clinical trials, and obtaining post clinical data are essential tasks.

In short, vaccination with inactivated vaccines is still an effective way to fight against the SARS-CoV-2 and variants epidemic.

## Data Availability

The results supporting the findings in this study are available upon request from the corresponding authors.
